# Synthesis, Antioxidant and Antimicrobial Activity of a New Phloridzin Derivative for Dermo-Cosmetic Applications

**DOI:** 10.3390/molecules171113275

**Published:** 2012-11-07

**Authors:** Anna Baldisserotto, Gemma Malisardi, Emanuela Scalambra, Elisa Andreotti, Carlo Romagnoli, Chiara Beatrice Vicentini, Stefano Manfredini, Silvia Vertuani

**Affiliations:** 1 Department of Life Sciences and Biotechnology, University of Ferrara, Via Fossato di Mortara 17-19, 44121 Ferrara, Italy; Email: bldnna@unife.it (A.B.); gemma.malisardi@unife.it (G.M.); emanuela.scalambra@unife.it (E.S.); chiara.vicentini@unife.it (C.B.V.); mv9@unife.it (S.M.); 2 Department of Life Sciences, University of Modena and Reggio Emilia, viale Caduti in Guerra 127, 41121 Modena, Italy; Email: elisa.andreotti@unimore.it (E.A.); carlo.romagnoli@unimore.it (C.R.); 3 Ambrosialab, Via Mortara 171, 44121 Ferrara, Italy

**Keywords:** phloridzin, F2, antioxidant activity, PCL, HPLC analysis, stability in dermocosmetic formulations, antifungal activity

## Abstract

The phenolic compound phloridzin (phloretin 2′-*O*-glucoside, variously named phlorizin, phlorrhizin, phlorhizin or phlorizoside) is a prominent member of the chemical class of dihydrochalcones, which are phenylpropanoids. Phloridzin is specifically found in apple and apple juice and known for its biological properties. In particular we were attracted by potential dermo-cosmetic applications. Here we report the synthesis, stability studies and antimicrobial activity of compound **F2**, a new semi-synthetic derivative of phloridzin. The new derivative was also included in finished formulations to evaluate its stability with a view to a potential topical use. Stability studies were performed by HPLC; PCL assay and ORAC tests were used to determine the antioxidant activity. **F2** presented an antioxidant activity very close to that of the parent phloridzin, but, unlike the latter, was more stable in formulations. To further explore potential health claims, antifungal activity of phloridzin and its derivative **F2** were determined; the results, however, were rather low; the highest value was 31,6% of inhibition reached by **F2** on *Microsporum canis* at the highest dose.

## 1. Introduction

Phloridzin (phloretin 2′-glucoside, phlorhizin, phlorrhizin) belongs to the chemical class of dihydrochalcones, phenylpropanoids with structures closely related to those of the immediate flavonoid precursors, the chalcones, and it is the major phenolic glucoside found in apple trees. Phloridzin has a bitter taste that contributes to the characteristic tang of cider [1], moreover the color of the apple juices derives from its dimerized oxidation products [2]. However, since its first isolation from the bark of apple trees in 1835 by De Koninck [3], phlorizin has attracted most scientific interest as a pharmaceutical and as a tool for human physiology research. 

Since year 2000 more than 700 peer reviewed articles, dealing with phloridzin or its derivatives, have appeared in literature. Its principal pharmacological action is to produce renal glycosuria and block intestinal glucose adsorption by inhibition of the sodium-linked glucose transporters. Most studies about phloridzin and its derivatives relate to antioxidant activity *in vitro*, with a wide range of biological related functions, such as inhibition of lipid peroxidation, prevention of bone loss, enhancement of memory and life extension as well as inhibition of cancer cell growth [4,5,6,7,8,9,10,11,12,13,14].

In contrast, knowledge about the physiological relevance of phloridzin in plants is limited. The biosynthetic steps leading to phloridzin were recently investigated with recombinant enzymes [15,16] and plant protein extracts [17].

Phloridzin not only exists in *Malus* species, in fact, phloretin glycosides have been founded in the leaves of Australian native sarsaparilla (*Smilax glyciphylla*) [18], sweet tea (*Lithocarpus polystachyus*) [19] and at very low levels in strawberry fruit [20]. In apple trees, phloridzin is found primarily in the young shoots, roots, leaves and bark, while in fruit, it is most abundant in the seeds, with intermediate levels in both the core and the skin, and the lowest level in the cortex. Despite this information, little is known of the phloridzin roles in plants (*i.e.*, apple tree physiology) although it has been suggested that it might act in apple tree growth and development [21] or as an inhibitor of bacterial [22] or fungal parasite growth [23].

It is well known that a diet high in fruits and vegetables decreases the risk of chronic diseases [24,25,26,27]. Most of the protective effects of fruits and vegetables have been assigned to phytochemicals, such as carotenoids, phenolics, flavonoids, and isoflavonoids which have different activities [28,29,30,31]. Phenylpropanoids are very interesting components among phenolics, but their dermo-cosmetic applications are very scarcely described in literature, we were thus attracted by the claimed photo-genoprotective effect of phloridzin-rich polyphenolic fractions [32]. The free-radical-scavenging mediated, anti-ultraviolet protective role of a phloridzin-rich polyphenolic fractions was measured on plasmid DNA. [33]. These effects, based on the antioxidant activity, are very interesting to the dermo-cosmetic field in view of the increasing attention of dermatologists to the solar DNA induced damages. In this application, the protective role was assessed against ultraviolet B rays in comparison to Parsol® MCX (2-methylhexyl *p*-methoxycinnamate). With an IC_50_ slightly less than Parsol® MCX, the polyphenolic extracts nevertheless showed large anti-ultraviolet B activity. 

Moreover, polyphenols-like structures also offer interesting dermo-cosmetic applications as potential antifungal agents, and this aspect has been also investigated in extracts from *Mallus* species [34]. Due to this latter information we also focused our attention on the antifungal activities of phloridzin. However, during preliminary investigations, in the aim to design topical formulations, important drawbacks were encountered in terms of very poor stability of phloridzin in finished formulations. The aglycon is much more easy to handle but it is not the natural form in which it is found in plants. In line with our recent strategy applied on other natural phenylpropanoids derivatives [35] we investigated novel phoridzin semi-synthetic derivatives in order to devise possible pro-drug forms. The latter were also investigated for stability in solutions at different pH and storage conditions and in finished dermo-cosmetic formulations. The antioxidant activity of the synthetic derivative **F2** and of the finished formulae containing it was determined by ORAC test and PCL assay, whereas antifungal activity was evaluated on nine dermatophytes in comparison between phloridzin and **F2**. The leading concept was that derivatization will increase the range of applications.

## 2. Results and Discussion

### 2.1. Chemistry

**Scheme 1:**
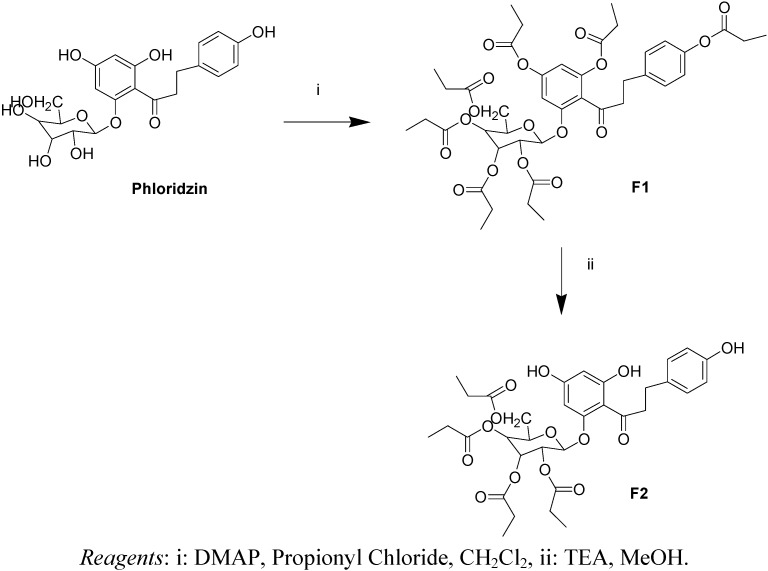
Synthesis of the tetra-substituted derivative of phloridzin **F2**.

Phloridzin was esterified at all hydroxyl moieties using an acyl chloride in presence of 4-dimethylaminopyridine (4-DMAP) and then selectively deprotonated at the phenolic hydroxyl groups, using N,N,N-triethylamine (TEA) in methanol (Scheme 1).

### 2.2. Stability Studies

**Figure 1:**
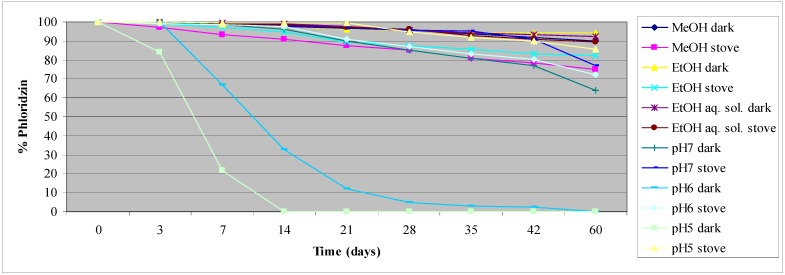
Phloridzin stability in solution at different pH values and storage conditions.

With the aim to evaluate the behaviour of phloridzin and **F2** as a function of time, pH and storage conditions, we conducted a stability study. Phloridzin was analyzed in MeOH, EtOH, EtOH–H_2_O solution (80:20, v/v) and in phosphate buffer solutions at pH 7, 6 and 5. All the solutions were stored at room temperature kept in the dark and subjected to accelerated aging at 40 °C in an oven. We have observed that phloridzin slowly precipitates at room temperature from the buffered solution at pH 5 over two weeks, whereas after 60 days in the oven, where solubility is complete, the recovery percentage is about 85.7%. This fact made it impossible to follow the stability at room temperature. The same behaviour was observed at pH 6. In all the other solutions, phloridzin was soluble and resulted stable with not too pronounced differences between room temperature and oven samples. We observed the best recoveries percentages of phloridzin over time (94%) in the sample in EtOH kept in the dark (Figure 1).

**Figure 2:**
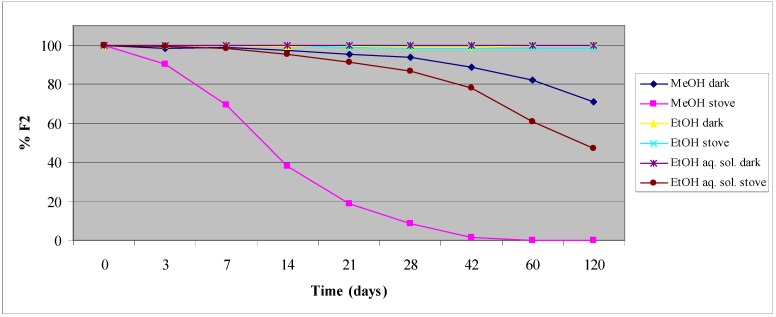
**F2** stability in different solutions under different storage conditions.

Regarding **F2**, we have evaluated MeOH, EtOH and EtOH-H_2_O solutions (80:20, v/v) as well. **F2** was not soluble at all in the buffer solutions used for the parent phlorizin study. Regarding the MeOH solutions, the results show that **F2** completely disappears after 6 weeks of accelerated aging conditions, with concomitant formation of phloridzin, while at room temperature and kept in the dark it was quite stable. In EtOH-H_2_O solution, after 120 days in the oven, **F2** hydrolyzed by 50%. In all the other solutions **F2** is stable (Figure 2).

In order to measure the lipophilic properties of the molecules, the partition coefficient (LogP), calculated as the logarithm of the ratio of concentrations of the substance in octanol and water, was evaluated. This determination was conducted in a biphasic system of octanol (20 mL) and water (20 mL at pH 6) at 25 °C. After 30 min of stirring, the biphasic mixture was centrifuged for 10 min at 4,000 rpm to complete separation of phases and then 5 mL of each phase was used for spectrophotometric determination (at 284 nm) concentrations of phloridzin (5 mg/20 mL) at the equilibrium.

A similar analysis has been performed for **F2** (5 mg/20 mL). The data (Table 1) shows a higher distribution in octanol than in water of phloridzin and **F2** (0.94 and 1.82 LogP respectively). These data confirm the greater lipophilicity of **F2** with respect to its parent compound phloridzin.

**Table 1:** Partition coefficients of phloridzin and F2 in a biphasic mixture of octanol and water.

	Octanol (mg/20 mL)	H_2_O (mg/20 mL)	LogP *
**Phloridzin**	4.48	0.52	0.94
**F2**	4.925	0.075	1.82

* LogP = log10 [mg/20 mL]octanol/[mg/20 mL]H_2_O.

### 2.3. Antioxidant Activity

**Figure 3:**
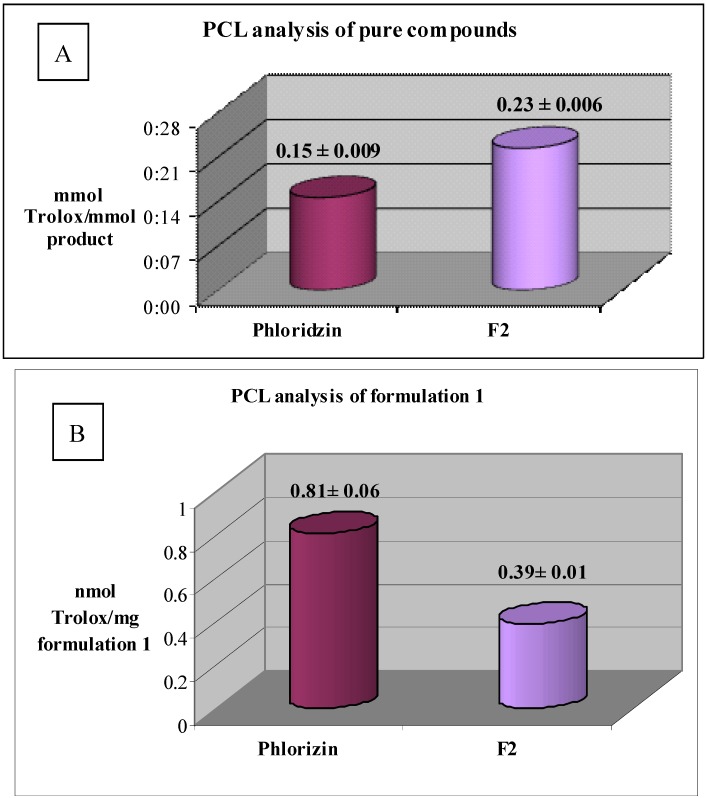
**Panel A**: PCL analysis of phloridzin and F2. **PanelB**: PCL analysis of phloridzin and F2 formulations. Each value was obtained from three experiments (Mean ± SE).

The two compounds were then tested to determine their antioxidant capacity by PCL analysis and ORAC test. PCL data showed similar antioxidant activity of the semi-synthetic derivative **F2** compared to that of phloridzin (0.23 and 0.15 mmol Trolox/mmol of product respectively) (Figure 3, Panel A). 

**Figure 4:**
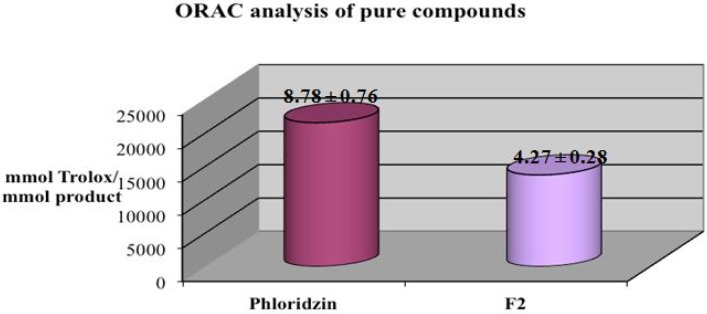
ORAC analysis of phloridzin and **F2**. Each value was obtained from three experiments (Mean ± SE).

Furthermore, the ORAC test results show a high antioxidant activity with respect to the peroxyl radical, with the best results seen for phloridzin as compared to **F2** (8.78 and 4.27 µmol Trolox/mmol product respectively) probably due to the fact that, being more lipophilic, within 24 h of analysis it might precipitate in the PBS (Figure 4).

### 2.4. Formulation Studies

Because the high potency of a molecule is useless if not expressed also within a formulation, we also investigated the antioxidant activity [36] and the stability of the active components incorporated into finished dermo-cosmetic formulations at 0.3% (p/p) and subjected to accelerated aging at 40 °C.

Two different cosmetic formulations, Formulation **1** (a cosmetic base O/W formula made with ingredients devoid of any antioxidant properties) and Formulation **2** (a water free formula also devoid of antioxidant properties) were designed.

PCL analysis (at time zero) has confirmed the antioxidant activity of the formulations containing phloridzin and **F2** as compared to the cosmetic base (0.81, 0.39 and 0 nanomol Trolox/mg formulation respectively) (Figure 3, Panel B).

The formulations were subjected to accelerated aging at 40 °C to monitor any changes in the products. This study (data not shown) showed that formulations containing phloridzin rapidly turned brown and casued separation in the O/W emulsion. Because this variation is not acceptable, we decided to focus our attention exclusively on the formulations containing **F2**.

**Figure 5:**
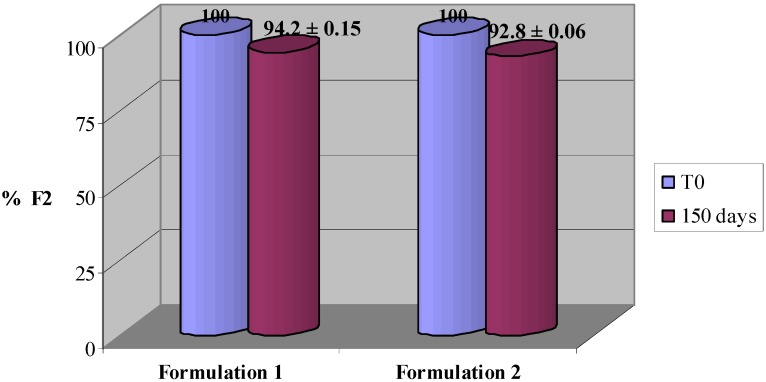
F2 recovery in the different formulations stored at 40 °C. Each value was obtained from three experiments (Mean ± SE).

PCL analysis of the formulation **1** containing **F2**, after 150 days at 40 °C, did not show any changes in the anti-oxidant activity. Formulations **1** and **2** containing **F2**, were also evaluated in parallel by HPLC to assess the stability in function of time. The analysis showed that F2 is stable in both the formulations **1** and **2** (Figure 5).

### 2.5. Antufungal Acitivity

Phloridzin and its derivative **F2** have been studied for their antifungal activities against nine fungal species [*Epidermophyton floccosum*(Hartz) Langeron e Milochevitch, *Trichophyton rubrum* (Castellani) Saboraud, *Trichophyton tonsurans* Malmsten, *Trichophyton violaceum*, *Trichophyton mentagrophytes* (Robin) Blanchard, *Microsporum gypseum* (Bodin) Guiart e Grigorakis, *Nannizzia cajetani* Ajello, *Microsporum canis* Bodin, *Nannizzia* *gypsea* (Nann.) Stockd. (perfect state of *Microsporum gypseum)*] using the Sabouraud Dextrose Agar (SDA) diffusion method, in DMSO solvent (Table 2).

As shown in Table 2, the antifungal activity of phloridzin and its derivative **F2**, did not give interesting outcomes: in fact, in all cases the percentages of inhibition are rather low and the highest value was 31.63% reached by **F2** on *Microsporum canis* at the highest dose. In other cases the substance showed an hormone-like effect with a fungal growth higher than that of the controls (+). This occurrence is so far unexplained and awaits future investigations about the possible mechanism of action: **F2** is mostly a pro-drug of phloridzin and should behave with the same mechanism.

**Table 2:** Growth inhibition following treatment with the two pure standards phloridzin and its derivative **F2**.

Fungus	% Growth inhibition on the 7th days by phloridzin conc. (μg/mL) of		% Growth inhibition on the 7th days by F2 conc. (μg/mL) of	
	20	100	20	100
*Epidermophyton floccosum*	+	+	+	+
*Trichophyton rubrum*	+	+	4.1 ± 0.3	4.0 ± 0.7
*Trichophyton tonsurans*	13.1 ± 1.1	7.1 ± 0.9	3.1 ± 0.4	8.0 ± 1.5
*Trichophyton violaceum*	0.7 ± 0.1	5.1 ± 1.2	20.1 ± 1.8	18.1 ± 1.7
*Trichophyton mentagrophytes*	+	+	22 ± 0.9	18.1 ± 0.7
*Microsporum gypseum*	4.1 ± 0.5	+	13.0 ± 1.2	16.0 ± 0.3
*Nannizzia cajetani*	+	+	9.1 ± 0.5	16.0 ± 1
*Microsporum canis*	+	7 ± 0.8	24.0 ± 1.1	31.6 ± 1.4
*Nannizzia gypsea*	0	+	11.0 ± 0.6	20.1 ± 0.8

## 3. Experimental

### 3.1. General

All reactives were from Sigma-Aldrch srl (Milan, Italy). Reaction course was routinely monitored by thin-layer chromatography on silica gel using precoated Macherey-Nagel Durasil-25 plates with detection under a 254-nm UV lamp and/or by spraying the plates with FeCl_3_ solution or potassium permanganate diluted solution. Column chromatography was performed with Macherey-Nagel 0.063–0.2 mm/70–230 mesh silica gel. The molecular weights of the compounds were determined by ESI (Micromass ZMD 2000), and the values are expressed as [MH]^+^. ^1^H-NMR spectroscopy was obtained using a Bruker AC-200, a Varian VXR-200 or a Mercury Plus400 spectrometer. HPLC analysis was performed using an Agilent 1100 Series HPLC System equipped with a G1315A DAD and with a Hydro RP18 Sinergi 80A column (4.6 × 150 mm, 4 μm) from Phenomenex. Phloridzin was purchased from Sigma Aldrich (Steinheim, Germany).

### 3.2. Synthesis

#### 3.2.1. Synthesis of phlorizin heptaproprionate (**F1**)

To a pre-cooled (0 °C) solution of phloridzin (400 mg, 0.92 mmol) and DMAP (4 g, 32.78 mmol) in CH_2_Cl_2_ (50 mL), propionyl chloride (19.32 mmol) was slowly added. After stirring at room temperature for 14 h the reaction mixture was washed with H_2_O, saturated NaHCO_3_ and with brine. The organic phase was then dried (Na_2_SO_4_), filtered and the solvent evaporated under reduced pressure to obtain an amber-colored oil. The residue was dissolved in Et_2_O and washed several times with water. The organic phase was then dried (Na_2_SO_4_), filtered and the solvent evaporated under reduced pressure to obtain a yellow oil. Crystallization with Et_2_O and petroleum ether, afforded **F1** (75% yield) as a white powder. ^1^H-NMR (400 MHz, CDCl_3_): δ (ppm) 1.163 (m, 21H, 7 × CH_3_); 2.417 (m, 14H, 7 × CH_2_); 2.913 (t, 2H, Ar-CH_2_-C*H_2_*-CO, *J* = 14.8 Hz); 2.989 (m, 1H, Ar-C*H_2_*-CH_2_-CO); 3.143 (m, 1H, Ar-C*H_2_*-CH_2_-CO); 3.90 (m, 1H sugar); 4.229 (m, 2H, -OCH_2_-aglycone); 5.022 (m, 1H sugar); 5.149 (m, 1H sugar); 5.276 (m, 1H sugar); 5.297 (m, 1H sugar); 6.684 (d, 1H aromatic, *J* = 2.2 Hz); 6.778 (d, 1H aromatic, *J* = 2.2 Hz); 6.987(d, 2H aromatic, *J* = 8.8 Hz); 7.247 (d, 2H aromatic, *J* = 8.4 Hz). ESI MS: *m/z* 829.7 Da [M+H]^+^, C_42_H_52_O_17_ Mol. Wt. 828,85.

#### 3.2.2. Synthesis of phlorizin tetraproprionate (**F2**)

Et_3_N (3.5 mL) was added to a solution of **F1** (501.8 mg, 0.61 mmol) in anhydrous MeOH (10.4 mL). The mixture was stirred at room temperature under argon atmosphere for 17 h and then neutralized by adding of formic acid (3 mL) at 0 °C. Next the mixture was evaporated under reduced pressure. The crude residue was dissolved in AcOEt and washed with water and brine. The organic phase was than dried (Na_2_SO_4_), filtered and the solvent evaporated under reduced pressure. The residue obtained was purified by silica gel column chromatography (eluent: CH_2_Cl_2_/MeOH, 98/2, v/v), gave **F2** (51%) as ivory foam. ^1^H-NMR (400 MHz, DMSO-*d_6_*): δ (ppm) 0.99 (m, 12H, 4xCH_3_); 2.22 (m, 8H, 4 × CH_2_); 2.699 (t, 2H, Ar-CH_2_-C*H_2_*-CO, *J* = 7.2 Hz); 2.924 (m, 1H, Ar-C*H_2_*-CH_2_-CO); 3.139 (m, 1H, Ar-C*H_2_*-CH_2_-CO); 4.046 (m, 1H sugar); 4.254 (m, 2H, -OCH_2_-aglycone); 5.02 (m, 1H sugar); 5.112 (m, 1H sugar); 5.453 (t, 1H sugar, *J* = 9.6 Hz); 5.693 (d, 1H sugar, *J* = 8 Hz); 5.984 (d, 1H aromatic, *J* = 2.2 Hz); 6.088 (d, 1H aromatic, *J* = 2.2 Hz); 6.621 (d, 2H aromatic, *J* = 8.8 Hz); 6.964 (d, 2H aromatic, *J* = 8.4 Hz); 9.11 (s, 1H, OH); 10.58 (s, broad, 1H, OH); 12.76 ( s, 1H, OH). ESI MS: *m/z* 661.5 Da [M+H]^+^, C_33_H_40_O_14_ Mol. Wt. 660,66.

### 3.3. Antioxidant Activity Assays

#### 3.3.1. Photochemiluminescence (PCL) Method

The PCL assay, based on the methodology of Popov and Lewin [37], was used to measure the antioxidant activity of extracts with a Photochem® apparatus (Analytik Jena, Leipzig, Germany) against superoxide anion radicals generated from luminol, a photo-sensitizer, when exposed to UV light (Double Bore® phosphor lamp, output 351 nm, 3 mWatt/cm^2^). The antioxidant activity was measured using both Antioxidant Capacity of Water (ACW) soluble substance and Antioxidant Capacity of Liposoluble (ACL) substance kits provided by the manufacturer designed to measure the antioxidant activity of hydrophilic and lipophilic compounds, respectively [38]. For ACW studies, the luminol reagent and Trolox work solution were freshly prepared according to the ACW protocol. The presence of Trolox (or any other antioxidants from the extracts) retarded luminescence for a period: hence, a lag time was noted before a signal was measured. The duration of the lag, which is calculated by the computer software from the first derivative of the detector signal at its turning point and intersection with the x-axis, was plotted against the concentration of Trolox added to the assay medium. The concentration of the added extract solution was such that the generated luminescence fell within the limits of the standard curve. Therefore, the lag time (seconds) for the ACW assay was used as the radical scavenging activity and the antioxidant capacity calculated by comparison with a Trolox standard curve and then expressed as Trolox equivalent. In ACL studies, the kinetic light emission curve, which exhibits no lag phase, was monitored for 180 s and expressed as micromoles of Trolox per gram of dry matter. The areas under the curves were calculated using the PCLsoft control and analysis software. As greater concentrations of Trolox working solutions were added to the assay medium, a marked reduction in the magnitude of the PCL signal and hence the area calculated from the integral was observed. This inhibition was used as a parameter for quantification and related to the decrease in the integral of PCL intensities caused by varying concentrations of Trolox. The observed inhibition of the signal was plotted against the concentration of Trolox added to the assay medium. The concentration of the added extract solution was such that the generated luminescence during the 180 s sampling interval fell within the limits of the standard curve. The extracts for ACW and ACL measurements were centrifuged (5 min at 16,000 g) prior to analysis. The antioxidant assay was carried out in triplicate for each sample, and 20 μL of the diluted extract (1:40, v/v) in HPLC-grade water (ACW) or HPLC-grade methanol (ACL) was sufficient to correspond to the standard curve. 

#### 3.3.2. Oxygen Radical Absorbance Capacity (ORAC) Assay

The ORAC assay was carried out on a Fluoroskan FL® ascent (Thermo Fisher Scientific, Inc. Waltham, MA, USA) with fluorescent filters (excitation wavelength: 485 nm; emission filter: 538 nm). The procedure was based on that given by Hong, Guohua & Ronald as modified in our previous work [39]. Briefly, in the final assay mixture (0.2 mL total volume), fluorescein sodium salt (85 nM) was used as a target of free radical attack with 2,2′-azobis(2-amidinopropane) dihydrochloride (AAPH) as a peroxyl radical generator. Trolox, a water-soluble analogue of vitamin E, was used as a standard control: a calibration curve was carried out with 10, 20, 30, 40, 50 μM solution. The tested compounds were dissolved in PBS and prepared immediately before the experiments. The fluorescence measurements, carried out at 37 °C, were recorded at 5 min intervals up 30 min after the addition of AAPH. The ORAC values, calculated as difference of the areas under the quenching curves of fluoresceine between the blank and the sample, were expressed as Trolox equivalents (TE), pH = 7.4. All the experiments were performed with three replicates.

### 3.4. Stability Studies

#### 3.4.1. Solution Preparation

The samples were prepared using phlorizin and **F2** in different aqueous solution. Phlorizin was solubilized in MeOH, EtOH, EtOH/H_2_O (80:20) and in buffer phosphate solutions with following pH values: 5, 6, 7. **F2** was dissolved in MeOH, EtOH and in EtOH/H_2_O (80:20) mixture. The samples were divided into two separate series, called “dark” (naturally preserved in the absence of light.) and “oven” (subjected to accelerated aging in an oven at 40 °C).

#### 3.4.2. Dermo-Cosmetic Formulations

In this study, **F2** in different formulations has been subjected to accelerated aging in an oven at 40 °C, and analyzed by HPLC with the aim to asses its content over time. The study was carried out on two different formulations, containing 0.3% of the active ingredient. The formulations tested are described following International Nomenclature of Cosmetic Ingredients (INCI) rules: (1) INCI: Aqua, Glycerin, Glyceryl stearate, Ceteareth-20, Ceteareth-12, Cetyl palmitate, Cetearyl alcohol, Dimethicone, Caprylic/capric triglyceride, Dicapryl carbonate, Symdiol 68T. From a technological point of view, it is an O/W formulation. (2) INCI: Nomcort T.I.O., Nomcort HK-G, Kester Wax K82D, Covabead LH170, denaturated EtOH. From a technological point of view it is an anhydrous formulation.

#### 3.4.3. HPLC Methods

HPLC analysis was performed using an Agilent 1100 Series HPLC System equipped with a G1315A DAD and with an Hydro RP18 Sinergi 80A column (4.6 × 150 mm, 4 μm) from Phenomenex. The mobile phase consisted of water (0.01 M H_3_PO_4_) (solvent A) and acetonitrile(0.01 M H_3_PO_4_) (solvent B).

(1) Phloridzin: in this study, phloridzin in solution has been subjected to accelerated aging in an oven at 40 °C, and analyzed by HPLC with the aim to asses the content of this active ingredient over time. The determination is carried out under isocratic condition, A (73%)/B (25%) Separation was monitorated with absorbance detection at 284 ± 8 nm. The flow rate was 1.0 ml/min, the injection volume was 2 μL and all separation was performed at 27 °C.

(2) **F2**: in this study, **F2** in solution and in formulations has been subjected to accelerated aging in an oven at 40 °C, and analyzed by HPLC with the aim to asses the content of this active ingredient over time. The determination is carried out under isocratic condition, A (40%)/B (60%). Separation was monitorated with absorbance detection at 284 ± 8 nm. The flow rate was 1.2 mL/min, the injection volume was 5 μl and all separation was performed at 27 °C.

#### 3.4.4. Statistical Evaluations

Relative standard deviations and statistical significance (Student’s t test; *p* ≤ 0.05) were given where appropriate for all data collected. One-way ANOVA and LSD *post hoc* Tukey’s honest significant difference test were used for comparing the bioactive effects of different samples. All computations were made using the statistical software STATISTICA 6.0 (StatSoft Italia srl, Vigonza (PD), Italy).

### 3.5. Antifungal Activity

#### 3.5.1. Microorganisms

Phloridzin and its derivative **F2** were tested on fungal species, pathogenic for animals and humans, such as some dermatophytes. The dermatophytes used were *Nannizzia cajetani* Ajello, CBS 495.70 strain; *Epidermophyton floccosum* (Hartz) Langeron and Milochevitch, CBS 358.93 strain; *Trichophyton violaceum* Malmsten, CBS 459.61 strain; *Trichophyton tonsurans* Malmsten, CBS 483.76 strain, *Trichophyton mentagrophytes* (Robin) Blanchard, CBS 160.66 strain, *Microsporum canis* Bodin CBS 4727 strain, * Nannizzia gypsea* (Bodin) Guiart et Grigoraki CBS 286.63 purchased from Centraal Bureau voor Schimmelcultures (CBS), Baarn, The Netherlands; *Trichophyton rubrum* (Castellani) Sabouraud IHME 4321 strain, *Microsporum gypseum* (Bodin) Guiart and Grigorakis IHME 3999 strain obtained from the Institute of Hygiene and Epidemiology-Mycology (IHME, Brussels, Belgium). The cultures were maintained in the laboratory as agar slants on a suitable culture medium, that is, on Sabouraud dextrose agar (SDA; Difco), for the dermatophytes.

#### 3.5.2. Evaluation of Antifungal Activity

To evaluate antifungal activity, cultures of each fungus were obtained by transplanting mycelium disks, 10 mm in diameter, from a single culture in stationary phase. These were incubated at 26 ± 1 °C on the medium suitable for each organism (SDA), on thin sterile sheets of cellophane, until the logarithmic phase of growth was reached. Then the fungi were transferred to Petri dishes containing the medium supplemented with the compound to be tested. Each compound was dissolved into dimethyl sulfoxide (DMSO), and a proper dilution was aseptically added to the medium at 45 °C to obtain a final concentration of 20, or 100 *μ*g mL^−1^. The DMSO concentration in the final solution was adjusted to 0.1%. Controls were set up with equivalent quantities (0.1%) of DMSO. The growth rate was determined by measuring daily colony diameter for 7 days after the transport of the fungus onto dishes containing the substance to be tested. At this time the percentage growth inhibition in comparison with the control was evaluated for each fungus. Three replicates were used for each concentration. The percentage of growth inhibition was expressed as the mean of values obtained in three independent experiments. 

The relative inhibition rate of the circle mycelium compared to blank assay was calculated via the following equation:

Relative inhibition rate (%) = [(dex − dex′)/dex] × 100%

where dex is the extended diameter of the circle mycelium during the blank assay; and dex' is the extended diameter of the circle mycelium during testing.

## 4. Conclusions

In conclusion, phloridzin and its derivative **F2** exhibited similar antioxidant capacity in PCL analysis and an ORAC test. This demonstrates that the structural modification does not decrease efficacy toward oxidative species. The importance of the structural modification is demonstrated by the formulation study, where the modification of the sugar hydroxyl groups increases lipophilicity, but keeps free the active phenol moieties, and allows one to obtain a molecule that is still active but much more stable in dermo-cosmetic formulae. On the other hand phloridzin itself is difficult to use because of the quick appearance of a brown coloration which is an undesirable behaviour for applications. In order to exploit functional effects, beside antioxidant ones, that could be useful for health applications, the antifungal activity of phloridzin and its derivative **F2** have been explored but they both show a low inhibition; only **F2** on *Microsporum canis* reached a remarkable value but significative enough to suggest a use as an antifungal. Interestingly, in all the other cases the substance showed an hormone-like effect with a fungal growth higher than that of the controls. This behavior certainly deserves further investigation. In order to explore the potential of phloridzin and its new derivative in the dermo-cosmetic and medicinal fields other studies are planned to investigate the antioxidant, anti-inflammatory, antiproliferative (on K562 and IB3-1 cells) activities.
